# From traditional remedy to *in vitro* evidence: phytochemical composition and skin-related pharmacological activity of birch witches’ broom (*Taphrina betulina*)

**DOI:** 10.3389/fphar.2026.1824563

**Published:** 2026-06-25

**Authors:** Adrian Strus, Weronika Skowrońska, Aleksandra Malarczyk, Maja Kirzyc, Jakub P. Piwowarski, Marcin Równicki

**Affiliations:** 1 Student Scientific Association “Herbarium”, Department of Pharmaceutical Biology, Faculty of Pharmacy, Medical University of Warsaw, Warsaw, Poland; 2 Department of Pharmaceutical Microbiology and Bioanalysis, Faculty of Pharmacy, Medical University of Warsaw, Warsaw, Poland; 3 Department of Pharmaceutical Biology, Faculty of Pharmacy, Medical University of Warsaw, Warsaw, Poland; 4 MicrobiotaLab, Department of Pharmaceutical Microbiology and Bioanalysis, Faculty of Pharmacy, Medical University of Warsaw, Warsaw, Poland

**Keywords:** *Betula*, ethnopharmacology, keratinocytes, Sámi, *Taphrina betulina*, witches’ broom

## Abstract

**Background:**

Birch witches’ broom is an abnormal plant structure caused by infection of birch trees with the dimorphic fungus *Taphrina betulina* Rostr., (1883). Although classified as a disease, witches’ broom has traditionally been used in folk medicine to treat skin disorders. This suggests that fungal infection may induce metabolic reprogramming in host tissues, leading to the production and accumulation of biologically active secondary metabolites. We hypothesized that infection-driven metabolic changes in witches’ broom tissue could generate compounds capable of modulating keratinocyte viability, migration, and inflammatory responses.

**Methods:**

Aqueous extracts from witches’ broom and healthy birch branches were analyzed by UHPLC-DAD-MS^n^. Biological activity was assessed *in vitro* using human keratinocytes (HaCaT) and melanoma cells (HTB-140). Cytotoxicity was determined by MTT assay, keratinocyte migration by scratch assay, pro-inflammatory cytokines IL-6/IL-8 by ELISA, and antimicrobial activity by disc diffusion against skin pathogens.

**Results:**

Phytochemical profiling revealed qualitative differences, particularly in flavonoids, phenolic acids, and diarylheptanoids. Cytotoxicity showed concentration-dependent effects. The IC_50_ values for witches’ broom and healthy birch extracts were 260.9 µg/mL and 327.1 µg/mL in HaCaT cells, and 487.5 µg/mL and 453 µg/mL in HTB-140 cells, respectively. Keratinocyte migration was examined using a scratch assay. After 48 h, witches’ broom extract inhibited HaCaT cell migration by 99.09% ± 4.71% at the highest tested concentration, compared to 84.33% ± 5.14% inhibition by the healthy birch extract. Witches’ broom extract significantly reduced IL-6 secretion in stimulated HaCaT cells, while showing limited effect on IL-8 production. Antimicrobial activity against skin-associated pathogens revealed selective *C. acnes* inhibition by witches’ broom extract.

**Summary:**

This study provides the first direct comparison of their phytochemical composition and skin-related biological activity. The findings suggest that witches’ broom contains bioactive compounds influencing cell viability, migration, and inflammation, supporting its potential relevance in dermatological research and ethnopharmacology.

## 1 Introduction

Plant-pathogen interactions are known to induce significant changes in host metabolism, frequently leading to the accumulation of secondary metabolites involved in plant defenses (Ullah et al., 2019; Gerber and Dubery, 2004). These infection-induced metabolic alterations often include enhanced production of phenolic compounds, flavonoids, and terpenoids, many of which exhibit biological activities of pharmacological relevance (Chen et al., 2022). In some cases, pathological plant tissues formed in response to infection may represent unique chemical groups characterized by metabolite profiles that differ substantially from those of healthy plant organs.

Birch witches’ broom is a distinctive morphological abnormality caused by infection with the dimorphic ascomycete fungus *Taphrina betulina* Rostr., (1883) (Christita et al., 2023; Christita et al., 2022). The disease is characterized by the formation of dense clusters of shortened, highly branched shoots that resemble a broom-like structure. This pathological growth results from complex interactions between fungal factors and host regulatory pathways, including hormonal imbalances and disruption of normal meristematic activity (Christita et al., 2023). While a witches’ broom is primarily considered a plant disease from a forestry and ecological perspective, ethnobotanical sources indicate that infected birch tissues were traditionally used in folk medicine for the treatment of skin disorders (DuBois and Lang, 2013; Liu-Helmersson and Ouma, 2021; Ouma et al., 2023; DuBois, 2011). In particular, historical accounts of Sámi ethnomedical practices describe the topical application of aqueous decoctions prepared from witches’ broom tissue to inflamed or diseased skin (DuBois and Lang, 2013).

Such traditional use suggests that infection-driven metabolic reprogramming in witches’ broom tissue may lead to the accumulation of compounds capable of modulating biological processes relevant to skin physiology. Fungal infection not only induces visible morphological changes in the host plant but also affects the biosynthesis of secondary metabolites. Previous studies on birch and related species have shown that pathogen-induced stress can modify the production of phenolic acids, flavonoids, triterpenes such as betulin, and diarylheptanoids (Smiljanic et al., 2022; Lee et al., 2012; Adepoju et al., 2023). These compound classes are widely recognized for their antioxidant, anti-inflammatory, and cytotoxic properties, as well as for their potential to influence cell proliferation and migration (Hayakawa et al., 2020; Chen et al., 2022).

Despite growing interest in plant defense chemistry and ethnopharmacology, scientific data on the chemical composition and biological activity of witches’ broom tissues remain scarce (Rose et al., 2025). Most phytochemical and pharmacological studies on birch have focused on healthy bark, leaves, or buds, which are well known sources of betulin, catechin derivatives, and other bioactive constituents (Smiljanic et al., 2022; Adepoju et al., 2023). In contrast, witches’ broom represents a metabolically altered plant material whose phytochemical profile may differ quantitatively and qualitatively from that of uninfected tissues. This raises the possibility that witches’ broom may contain unique or enriched metabolites with distinct biological activities.

From a dermatological perspective, keratinocytes play a central role in maintaining skin barrier function, wound healing, and inflammatory signaling (Guo and Dipietro, 2010; Takada et al., 2017). Dysregulation of keratinocyte proliferation, migration, or cytokine secretion contributes to the pathogenesis of numerous skin disorders, including atopic dermatitis and psoriasis (Tampa et al., 2022). Interleukin-6 (IL-6) and interleukin-8 (IL-8) are key mediators of cutaneous inflammation and immune cell recruitment, and their excessive production is associated with impaired wound healing and chronic inflammatory states (Takada et al., 2017). Therefore, compounds capable of modulating keratinocyte behavior and cytokine release are of considerable interest for skin-related research.

In this study, we investigated whether the traditional use of witches’ broom for skin applications is supported by scientific evidence. To preserve ethnobotanical authenticity, aqueous extracts obtained from witches’ broom and healthy birch tissues were subjected to phytochemical profiling and evaluated using *in vitro* models of human skin cells (Colombo et al., 2017). We focused on key biological endpoints relevant to dermatological conditions, including cell viability, keratinocyte migration, secretion of pro-inflammatory cytokines (IL-6 and IL-8), and antimicrobial activity. Together, this approach provides a scientifically grounded evaluation of witches’ broom as a potential source of bioactive compounds relevant to dermatological applications.

This study contributes to ethnopharmacology by examining birch witches’ broom, a material recorded in Sámi traditional medicine as a topical treatment for skin disorders (DuBois, 2011; Liu-Helmersson and Ouma, 2021). By focusing on a pathological plant structure formed through fungal infection, it broadens ethnopharmacological research beyond typical medicinal plant organs (Yeung et al., 2020; Pirintsos et al., 2022; Heinrich, 2015). The comparison of infected and healthy birch tissues helps assess whether traditional selection was linked to altered phytochemical composition and biological activity (Ullah et al., 2019). In this way, the study contributes to a more critical and evidence-based evaluation of traditional dermatological practices (World Health, 2019; Ouma et al., 2023).

## 2 Materials and methods

### 2.1 Plant material

Witches’ broom samples were collected from naturally infected birch tree (*Betula* spp.) in Berlin, Germany, between November 2023 and April 2024 (exact location 52.657771185° N, 13.3087372407° E). Control (healthy) birch branches were collected from a different birch tree located within the same sampling area and lacking visible witches’ broom symptoms. All material used for extract preparation and subsequent analyses was obtained from Berlin to ensure experimental consistency and minimize environmental variability. This limitation should be considered when interpreting the results, as metabolite composition in plant tissues may vary depending on climate, season, and local environmental conditions.

Host plant material was authenticated based on macroscopic morphological characteristics, and a voucher specimen (voucher no. 3011/23) has been deposited in the Department of Pharmaceutical Biology, Medical University of Warsaw, Poland. The presence of *T. betulina* Rostr., (1883) in witches’ broom samples was confirmed by polymerase chain reaction (PCR) analysis targeting the internal transcribed sequence (ITS) region of fungal DNA according to protocol by Christita et al. (2022). Collected samples were air-dried at room temperature under dark conditions to prevent photodegradation of metabolites and subsequently ground into a fine powder using a laboratory mill.

### 2.2 Extract preparation

Dried witches’ brooms and healthy birch branch material were extracted threefold with boiling distilled water at a 1:10 (w/v) ratio over 30 min in an ultrasonic bath, approximating traditional aqueous decoctions used in folk medicine. The extracts were subsequently freeze-dried, stored at 4 °C in the dark until further analysis. Extraction yield, calculated relative to the dry weight of the starting material, was 20.7% (w/w) for healthy birch and 15.6% (w/w) for witches’ broom.

### 2.3 Phytochemical profiling

Phytochemical profiling was carried out using an ultra-high-performance liquid chromatography equipped with a diode array detector and coupled to an ion trap mass spectrometer (UHPLC-DAD-MS^n^) via an electrospray ionization interface. UV-Vis spectra were recorded in the range of 200–450 nm. The mass spectrometer was operated under the following conditions: nebulizer pressure of 40 psi, drying gas flow rate of 9 L/min, nitrogen gas temperature of 134 °C, and capillary voltage of 4.5 kV. Mass spectra were acquired over a scan range of 70–2,200 m/z. The mobile phase consisted of solvent A (H_2_O/formic acid; 100:0.1, v/v) and solvent B (acetonitrile/formic acid; 100:0.1, v/v). Chromatographic separation was achieved on a reversed-phase C18 column (Kinetex XB-C18, 150 mm × 3.0 mm, 2.6 µm particle size) maintained at 25 °C. The gradient elution program was: 0–60 min 1%–26% B, 60–70 min 26%–40% B. The flow rate was maintained at 0.3 mL/min and the injection volume was 10 µL. Aqueous extracts were filtered through a 0.45 μm PVDF syringe filter and analyzed at a concentration of 10 mg/mL. Compounds were tentatively classified based on retention times and mass spectral data, in comparison with published literature and available databases, without confirmation using reference standards. Mass spectra relied on ESI in negative ion mode. In addition, crude extracts were analyzed after polyvinylpolypyrrolidone (PVPP) treatment to reduce interference from high-molecular-weight polyphenols, using the same UHPLC-DAD-MS^n^ conditions as for untreated extracts.

### 2.4 Cell culture conditions

Human immortalized keratinocytes (HaCaT) and human immortalized melanoma cells (HTB-140) were cultured in Dulbecco’s Modified Eagle Medium (DMEM) supplemented with 10% fetal bovine serum (FBS) and 1% penicillin-streptomycin solution. Cells were maintained at 37 °C in a 90% humidified incubator with 5% CO_2_. The medium was changed every 2–3 days, and cells were subcultured at approximately 80%–90% confluence using trypsin-EDTA solution.

#### 2.4.1 Cell viability assay (MTT)

Cell viability was assessed using the MTT reduction assay. Both HaCaT cells and HTB-140 cells were seeded separately into a 48-well plate at a density of 2 × 10^5^ cells/well and incubated for 24 h (corresponding to approximately 80% confluence at the time of treatment). Cells were then treated with healthy birch and witches’ broom extracts at various concentrations (15.63 µg/mL, 31.25 µg/mL, 62.5 µg/mL, 125 µg/mL, 250 µg/mL, 500 µg/mL, 1 mg/mL, 2 mg/mL, and medium as control) and incubated for another 24 h. After treatment, the cells were washed with Dulbecco’s Phosphate Buffered Saline (DPBS) and 300 μL of MTT solution (0.5 mg/mL) was added to each well. Cells were then incubated for an additional 1 h at 37 °C. The medium was removed, and formazan crystals were dissolved in 300 μL DMSO. After complete solubilization, 100 µL of each sample was transferred to a 96-well plate for absorbance measurement at 570 nm and 630 nm using a microplate reader (SYNERGY™ 4, BioTek, Winooski, VT). Cell viability was calculated as a percentage relative to untreated control cells. 0.1% Triton X-100 in DPBS was used as a positive control.

#### 2.4.2 Cell migration (scratch) assay

The cell migration (scratch assay) was performed according to the previously published protocol (LiangPark and Guan, 2007). Briefly, HaCaT cells were seeded into 12-well plates at a density of 2 × 10^6^ cells per well and cultured for 18 h. The medium was replaced with serum-free DMEM for 4 h to minimize proliferation-related effects. A linear scratch was generated using a sterile 200-µL pipette tip. Detached cells were removed by washing with DBPS. Fresh serum-free medium containing plant extracts at final concentrations of 15.63, 31.25, or 62.5 µg/mL was added. Control wells received medium only. Images were captured at 0, 4, 20, 28, and 48 h using an inverted microscope equipped with a digital camera (Nikon Eclipse TS100, Nikon Instruments Inc., Melville, NY, United States, supported by NIS-Elements BR 3.22 Software). Scratch area was quantified using image analysis software. Scratch closure was first calculated as the percentage reduction in wound area relative to baseline, according to the following formula:

$$\text{scratch closure }\left(\%\right)=\frac{A_{0}-A_{t}}{A_{0}}\times 100$$

where *A*_*0*_ is the scratch area at 0 h and *A*_*t*_ is the scratch area at the corresponding time point. The inhibitory effect of the extracts on cell migration was then expressed as percentage inhibition relative to the untreated control, according to the following formula:

$$\text{migration inhibition }\left(\%\right)=\frac{C-T}{C}\times 100$$

where C is the percentage of scratch closure in the untreated control and T is the percentage of scratch closure in extract-treated cells at the corresponding time point.

### 2.5 Cytokine measurement (IL-6 and IL-8)

Cells were seeded into 24-well plate and incubated to approximately 80-90% confluences, 580 µL of medium containing witches’ broom or healthy birch extracts was added at final concentrations of 7.81 µg/mL, 15.63 µg/mL, 31.25 µg/mL, and 62.5 µg/mL. After 4 h of pre-incubation with the extracts, cells were stimulated with 20 µL of a cytokine mixture containing TNF-α and IFN-γ, each at a final concentration of 10 ng/mL. Levels of IL-6 and IL-8 were determined using ELISA kits in cell supernatants after 20 h of stimulation, in accordance with the manufacturer’s instructions (BD Biosciences. Human IL-6 ELISA Set (Cat. No. 555220) and BD Biosciences. Human IL-8 ELISA Set (Cat. No. 555244). Absorbance was measured using a microplate reader (SYNERGY™ 4, BioTek, Winooski, VT) at two wavelengths λ = 450 nm and λ = 570 nm.

### 2.6 Antimicrobial activity

Antimicrobial activity was evaluated using the disc diffusion method on Brain Heart Infusion Agar following standard procedures for inoculum preparation and incubation. The following reference bacterial strains were tested: *Cutibacterium acnes* ATCC 11827; *Staphylococcus aureus* ATCC 29231; *Staphylococcus epidermidis* ATCC 12228; *Micrococcus luteus* ATCC 10240; *Escherichia coli* ATCC 25922. Bacteria were incubated for 24–48 h at 35 °C (for *C. acnes*, anaerobic conditions were used). A clinical isolate *Candida albicans* MK25 was grown for 48 h at 30 °C. After incubation, fresh inocula were prepared in sterile 0.9% NaCl and adjusted to 0.5 McFarland standard turbidity and then applied to the entire Petri dish using a sterile swab. Paper disks were soaked with 20 µL of 50 mg/mL of healthy birch or witches’ broom extract (final amount of extracts per disc 1 mg) and then placed equidistantly on the dish. For bacterial strains, ciprofloxacin 5 µg disks (Oxoid, United Kingdom) were used as a positive control, whereas amphotericin B 20 µg disks (Sigma-Aldrich, Germany) served as a positive control for *C. albicans*; disks impregnated with DMSO were used as a negative control. Plates were incubated at 35 °C for 24 h for bacteria and at 30 °C for 24 h for *C. albicans*, under appropriate aerobic or anaerobic conditions as required by each microorganism. After incubation, the diameters of the zones of growth inhibition around each disk were measured in millimeters.

### 2.7 Statistical analysis

All experiments were performed in at least two independent replicates. Data are presented as mean ± standard deviation (SD) or standard error of the mean (SEM). Statistical analysis was performed using GraphPad Prism software (version 8.0.1, GraphPad Software, United States). Comparisons between groups were conducted using one-way analysis of variance (ANOVA). A value of *p* < 0.05 was considered statistically significant.

## 3 Results

### 3.1 Phytochemical profile

**FIGURE 1:**
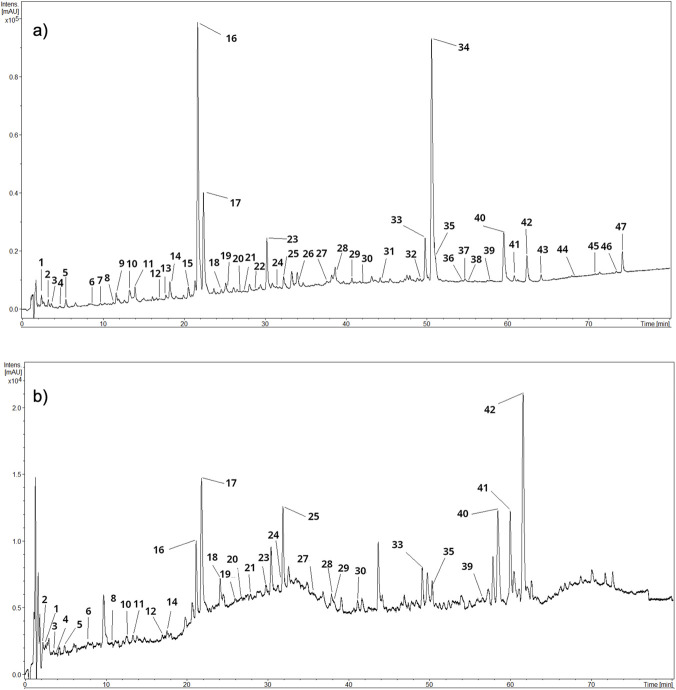
Chromatographic profiles of the **(a)** witches’ broom and **(b)** healthy birch crude aqueous extracts recorded by UHPLC-DAD-MSn at 190–450 nm.

Phytochemical analysis of witches’ broom extract revealed features consistent with several classes of secondary metabolites, including flavonoids (predominantly catechin derivatives), phenolic acids, arylbutanoids, and diarylheptanoids (Lee et al., 2012; Liimatainen et al., 2012; Smiljanic et al., 2022). The chromatographic profile of the witches’ broom extract (Figure 1a) differed from that of extract obtained from control healthy birch material (Figure 1b). The major detected compounds are listed in Table 1.

**TABLE 1:** Phytochemical profile of witches’ broom extract determined by UHPLC-DAD-MS^n^.

Peak no.	Rt [min]	UV-Vis max [nm]	MS (-)	MS^2^	Assignment	Literature
1	2.6	190	191, **436**	111, 173, 169, 199, 257, 417, 392, 332, 301	Isocitric acid	Affes et al. (2021)
2	3.3	198	**606**	385, 282, 403, 362, 323, 273, 211, 177, 508, 563	Unknown	—
3	3.9	190	317, **611**	306, 482, 592, 338, 272, 254, 210	Arbutin	Repert et al. (2022)
4	4.9	190	297, **607**	563, 545, 527, 491, 411, 437, 399, 257, 215	Unknown	—
5	5.7	190	707, **737**	423, 449, 555, 361, 379, 287, 257	(epi)gallocatechin	Cádiz-Gurrea et al. (2014)
6	8.6	190	**493**	331, 169	Monogalloyl dihexoside	Soong and Barlow (2005)
7*	9.9	203	**421**	357, 327, 195, 165	Loganic acid	Kucharska and Fecka (2016)
8	11.4	207	386, **575**	407, 513, 449, 423, 325, 289, 557	Procyanidin A	Soong and Barlow, (2005)
9*	11.8	205	**315**, 631	153, 369	Protocatechuic acid O-hexoside	Salomon et al. (2021)
10	13.5	201	432, **865**	740, 567, 407, 289, 450, 414	Procyanidin C	Zhang et al. (2023)
11	14.2	201	**498**, 997	422, 289, 245, 331, 167, 448, 432, 406	Procyanidin C O-xyloside	Liimatainen et al. (2012)
12	17.1	207	**447**, 613	177, 207, 263, 315	Catechin O-dihexoside	Xiang et al. (2019)
13*	17.6	209	**351**, 703	249, 289, 99, 143, 125, 161	Catechin derivative	Zhang et al. (2024)
14	18.5	206	**461**, 923	315, 415, 163	Kaempferol-O-glucuronide	Gouveia-Figueira and Castilho (2015)
15*	20.8	204, 276	**443**, 327	352, 219, 143, 395, 161, 189, 201, 237, 425	Unknown	—
16	21.9	199, 278	**421**, 843	289, 245, 137	Catechin O-pentoside	Fernandes et al. (2024)
17	22.6	201, 278	289, **579**	288, 245	Catechin adduct	Kajdžanoska et al. (2010)
18	24.8	207, 277	513, **526**	477, 293	Diarylheptanoid glucoside	Felegyi-Tóth et al. (2022)
19	25.4	206	431, **507**	345, 489, 241, 327	Unknown	—
20	26.7	209	421, **431**	385, 205, 153	Catechin O-pentoside	Xiang et al. (2019)
21	27.1	209, 275	405, **431**	179, 385, 225, 297, 205, 161, 134	Caffeic acid derivative	Zhu et al. (2015)
22*	28.3	209	**345**, 691	271, 221, 225, 211, 159, 100	Unknown	—
23	30.4	190, 276	**373**, 655	327	Rhododendrin	Smiljanic et al. (2022)
24	31.1	209	**443**, 459, 509	425, 143, 178, 263, 327, 165	Apiosylrhododendrin	Smiljanic et al. (2022)
25	32.5	191	**459**, 505	327, 165	Apiosylrhododendrin	Smiljanic et al. (2022)
26*	34.2	209, 270	**343**, 687	211, 269, 313, 449	Unknown	—
27	36.2	210	459, **469**	423, 293, 339, 149	Unknown	—
28	38.9	206	**521**, 597	475, 313	Platyphylloside	Lee et al. (2012)
29	40.9	209	511, **521**	475, 313	Platyphylloside	Lee et al. (2012)
30	41.9	212	**471**, 449	328, 309, 370, 409	Apigenin derivative	Ali et al. (2021)
31*	44.4	211	523, **569**	523, 361	Unknown	—
32*	49.1	212	509, **519**	473, 306, 353, 163	Unknown	—
33	49.9	192, 213	607, **653**	607, 310	Unknown	—
34*	50.8	194, 220, 277	294, 475, **521**, 951	475, 295	Platyphylloside	Lee et al. (2012)
35	51.1	191, 215, 277	475, **521**, 951	475, 295	Platyphylloside	Lee et al. (2012)
36*	54.2	213, 275	491, **495**	167, 449	Unknown	—
37*	54.5	213	**495**, 787	447, 167, 415, 625, 329, 269	Unknown	—
38*	55.0	214	315, **505**	459, 297	Unknown	—
39	57.7	214, 277	**481**, 619	315, 153, 411, 295, 475, 189	Unknown	—
40	59.6	191, 216, 277	313, **627**	465, 149, 295	Unknown	—
41	61.0	214	**725**, 771	**593**, 461, 299, 673, 751, 464	Diapiosyl-O-aceroside	Lee et al. (2012)
42	62.6	192, 215	**593**, 639	**461**, 299	Diapiosyl-O-aceroside	Lee et al. (2012)
43*	64.2	215	461, **507**	299, 461	Aceroside VIII (adduct)	Lee et al. (2012)
44*	67.6	215	**327**, 655	229, 171, 291	Oxodihydroxy-octadecenoic acid	Llorent-Martínez et al. (2015)
45*	70.6	215	**329**, 659	211, 311, 229, 293, 183, 165, 113	Unknown	—
46*	71.4	215	**608**, 653	607, 475, 313	Diarylheptanoid glycoside	Felegyi-Tóth et al. (2022)
47*	74.3	192, 216	**295**, 591	189	Unknown	—

Tentative assignment of constituents was achieved through analysis of chromatographic retention times, UV-Vis spectra, and ESI mass spectra acquired in negative ion mode, with structural assignment supported by MS^2^ fragmentation patterns and comparison with previously reported data and spectral databases. Base peak mark in bold. Asterisks (*) indicate compounds unique to witches’ broom.

A qualitative analysis using UHPLC-MS was performed to characterize and compare the phytochemical profiles of witches’ broom and healthy birch tree extracts. In total, 47 chromatographic peaks were observed in witches’ broom chromatogram (Table 1). Of these, 30 peaks were tentatively annotated based on matches to available databases (Reaxys) and literature (supported by MS data). Among them, 17 infection-associated secondary metabolites were selectively detected in witches’ broom (Table 1, marked with asterisks). These included loganic acid (peak 7), protocatechuic acid 4-O-hexoside (peak 9), a catechin derivative (peak 13), diarylheptanoid-related features (peaks 34, 43, and 46), oxodihydroxy-octadecenoic acid (peak 44), and several unresolved peaks (15, 22, 26, 31, 32, 36, 37, 38, 45, 47). Multiple catechin/procyanidin-related peaks and birch-characteristic metabolites were shared between witches’ broom and healthy birch samples (Table 1).

Following this structural classification, the catechin-based compounds were considered first. Compounds 8, 10, and 11 were annotated as procyanidins and related derivatives, as indicated by fragmentation to m/z 289, corresponding to the catechin anion. Compound 8 was assigned as a type A procyanidin (Soong and Barlow, 2005), showing m/z 575 [M-H]^-^ and MS^2^ fragments at m/z 557 [M-H-H_2_O]^-^, m/z 513 [M-H-H_2_O-CO_2_]^-^, m/z 289 (from catechin), m/z 423 (retro-Diels-Alder (RDA) cleavage), and m/z 449 (heterocyclic ring fission (HRF) pathway). Compound 10, on the other hand, was annotated as a catechin trimer (type C1 procyanidin) (Zhang et al., 2023). It exhibits m/z 865 [M-H]^-^ and MS^2^ fragments at m/z 576, 740, and 450, which correspond to the expected fragments m/z 577, 739, and 451 for type C1 procyanidins considering the interflavan bonds, as well as m/z 289 [M-H]^-^. Compound 11 was likewise assigned as a trimeric catechin derivative, but modified by a pentose residue, most likely xylose, attached to a hydroxyl group of the catechin subunit (Liimatainen et al., 2012). It exhibited m/z 997 [M-H]^-^ and MS^2^ fragments at m/z 498 [M-2H]^2-^. Based on literature data, this compound would be expected to show m/z 332 [M-3H]^3-^, which is not observed in the fragmentation. This assumption is consistent with the observed fragmentation at m/z 289 [M-H-Pentose]^-^, originating from the catechin unit. Compounds 12, 16, and 20 were also putatively classified as catechin derivatives. Compound 12 was annotated as a catechin O-dihexose (Xiang et al., 2019). It exhibited m/z 613 [M-H]^-^, with MS^2^ fragments at m/z 451 [M-H-Hexose]^-^ and m/z 289 [M-H-Hexose-Hexose]^-^. Compounds 16 and 20, on the other hand, were assigned as catechin O-pentose derivatives (Xiang et al., 2019), with compound 16 present as an adduct (Fernandes et al., 2024). They were observed as m/z 843 [2M-H]^-^ and m/z 421 [M-H]^-^, with MS^2^ fragments at m/z 289 [M-H-Pentose]^-^. Compound 13 is most likely a catechin derivative, based on the presence of the characteristic m/z 289 fragment, typical of catechin. This hypothesis is further supported by the fact that both preceding and subsequent compounds are also catechin derivatives. Similarly, compound 17 is also considered a catechin derivative for the same reason as compound 13. It exhibits the characteristic m/z 289 fragment and additionally shows m/z 579, which may indicate the formation of an adduct from two catechin molecules.

Hydroxybenzoic acids were represented by compound 6 and compound 9. Compound 6 was annotated as a monogalloyl dihexoside (Soong and Barlow, 2005). It exhibited m/z 493 [M-H]^-^, with MS^2^ fragments at m/z 331 [M-H-Hexose]^-^ and m/z 169 [M-H-Hexose-Hexose]^-^ (corresponding to the gallic acid anion). Compound 9 was annotated as a O-hexose protocatechuic acid (adduct) (Salomon et al., 2021), based on m/z 631 [2 M-H]^-^ and m/z 315 [M-H]^-^, as well as MS^2^ fragments at m/z 297 [M-H-H_2_O]^-^, m/z 153 [M-H-Hexose]^-^, and m/z 108 [M-H-Hexose-CO_2_]^-^.

Compounds 23, 24, and 25 share a common 4-(4-hydroxyphenyl)-butan-2-ol skeleton, supporting their assignment to the arylbutanoid class (Smiljanic et al., 2022). Compound 23 was annotated as rhododendrin (betuloside), based on m/z 655 [2 M-H]^-^ and m/z 373 [M + HCOO]^-^, with MS^2^ fragments at m/z 327 and m/z 165. Compounds 24 and 25 exhibited m/z 505 [M + HCOO]^-^ and m/z 459 [M-H]^-^, with MS^2^ fragments at m/z 327 and m/z 165, and were annotated as apiosylrhododendrin.

Compounds 28, 29, 34, and 35 were annotated as platyphylloside, belonging to the class of diarylheptanoids (Lee et al., 2012). They were observed as m/z 521 [M + HCOO]^-^, with MS^2^ fragments at m/z 475 [M-H]^-^, m/z 313 [M-H-Hexose]^-^, and m/z 295 [M-H-Hexose-H_2_O]^-^. Compound 34 was detected as an adduct (two molecules of platyphylloside). Compounds 41, 42, and 43 were annotated as derivatives of aceroside VIII (Lee et al., 2012). Compound 41 was observed at m/z 771 [M + HCOO]^-^, with MS^2^ fragments at m/z 725 [M-H]^-^, m/z 593 [M-H-Pentose]^-^, m/z 461 [M-H-Pentose-Pentose]^-^, and m/z 299 [M-H-Pentose-Pentose-Hexose]^-^. This fragmentation pattern strongly indicates diapiosyl-O-aceroside VIII. Similar fragmentation pattern was observed for compound 42, except that m/z 771 and m/z 725 were absent, suggesting the loss of one apiose unit, and thus the compound was annotated as apiosyl-O-aceroside VIII. Compound 43 was observed as m/z 923 [2 M-H]^-^, with MS^2^ fragments at m/z 461 [M-H-Pentose]^-^ and m/z 299 [M-H-Pentose-Hexose]^-^, indicating the formation of an adduct composed of two molecules of aceroside VIII.

Compound 7, belonging to the class of iridoids, was annotated as loganic acid (Kucharska and Fecka, 2016). It was observed at m/z 421 [M + HCOO]^-^, with MS^2^ fragments at m/z 375 [M-H]^-^, m/z 357 [M-H-H_2_O]^-^, m/z 195 [M-H-H_2_O-Hexose]^-^, and m/z 165 [M-H-H_2_O-Hexose-HCOH]^-^.

Compound 21 was tentatively classified as a hydroxycinnamic acid derivative, consistent with caffeic acid fragmentation (Zhu et al., 2015). Based on the literature, the observed fragmentation matches the reported compound, showing m/z 179, 297, 161, and 134.

Additional putatively identified organic acids include compounds 1 and 44. Compound 1, with m/z 191 [M-H]^-^, showed MS^2^ fragmentation at m/z 173 [M-H-H_2_O]^-^ and m/z 111. It was classified as isocitric acid (Affes et al., 2021). Its initial m/z is identical to quinic acid (m/z 191 [M-H]^-^ and m/z 173 [M-H-H_2_O]^-^). The key clue for annotation was the fragment m/z 111 [M-H-H_2_O-CO_2_]^-^ distinguishing it as isocitric acid. Compound 44 was observed at m/z 655 [2M-H]^-^ and m/z 327 [M-H]^-^, with MS^2^ fragments at m/z 229, m/z 171, and m/z 291. It was identified as oxodihydroxy-octadecenoic acid (Llorent-Martínez et al., 2015). Although its initial fragmentation resembled that of platyphylloside, this possibility was excluded based on the subsequent MS^2^ fragmentation pattern.

**FIGURE 2:**
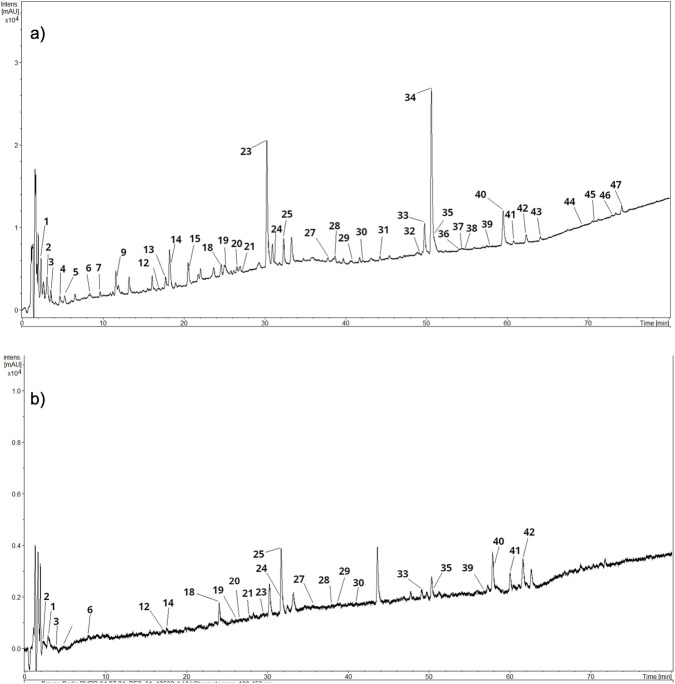
Chromatographic profiles of **(a)** PVPP-treated witches’ broom and **(b)** PVPP-treated healthy birch recorded by UHPLC-DAD-MSn in 190–450 nm.

In addition, UHPLC-MS analyses were performed on PVPP-treated extracts to aid compound annotation by reducing signals from high-molecular-weight polyphenols; the resulting chromatograms are presented in Figure 2a for witches’ broom and Figure 2b for healthy birch. PVPP treatment did not reveal additional metabolite classes, but by reducing signals from abundant catechin-rich polyphenols it made the existing differences between healthy birch and witches’ broom extracts more apparent, particularly the presence of diarylheptanoid-, arylbutanoid-, and organic acid-related features in the infected sample.

### 3.2 Cell viability assay (MTT)

**FIGURE 3:**
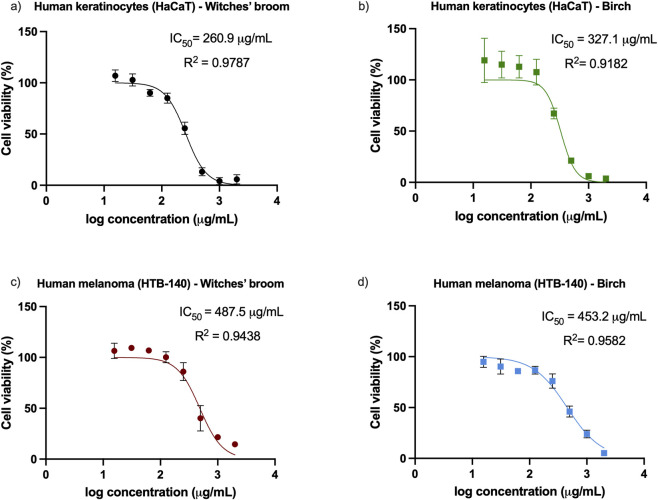
Cell viability measured by MTT assay after 24 h treatment with **(a)** witches’ broom extract in HaCaT cells, **(b)** healthy birch extract in HaCaT cells, **(c)** witches’ broom extract in HTB-140 cells, and **(d)** healthy birch extract in HTB-140 cells. Data are presented as mean ± SD from three independent experiments (n = 3).

The cytotoxic effects of witches’ broom extract and healthy birch extract were evaluated using the MTT assay. Both extracts reduced cell viability in a concentration-dependent manner in HaCaT and HTB-140 cell lines (Figure 3).

Notably, keratinocytes were more susceptible to both extracts than melanoma cells, as reflected by lower IC_50_ values in HaCaT (260.9 µg/mL for witches’ broom and 327.1 µg/mL for healthy birch, Figures 3a, b) compared with HTB-140 (487.5 µg/mL and 453 µg/mL, respectively) (Figures 3c, d). This difference indicates that the aqueous preparations exert stronger effects on non-transformed epidermal cells than on the tested melanoma cells under the present conditions. Accordingly, the extracts cannot be considered selectively cytotoxic toward melanoma, and the greater sensitivity of keratinocytes should be regarded primarily as a safety-relevant finding, as excessive toxicity toward epidermal cells could impair barrier integrity and re-epithelialization *in vivo*. Based on the viability results, only concentrations that did not significantly reduce HaCaT cell viability compared to untreated controls were selected for the cell migration assay and cytokine measurements.

### 3.3 Cell migration (scratch) assay

**FIGURE 4:**
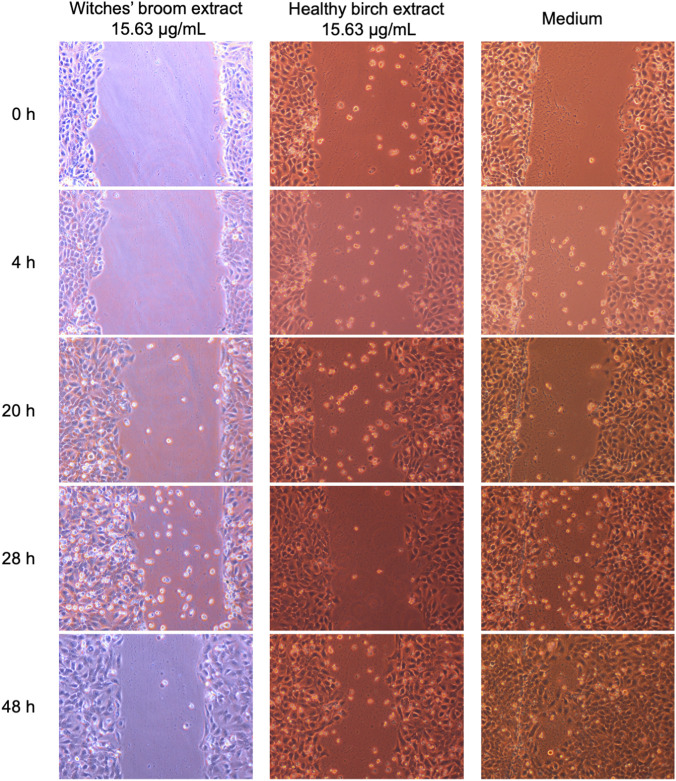
Representative images illustrating the effect of witches’ broom extract and healthy birch extract on HaCaT keratinocyte migration in the scratch assay in a concentration 15.63 µg/mL. Images were recorded at 0, 4, 20, 28, and 48 h after scratching. This figure shows representative images; quantitative results are presented in Table 2 as mean ± SD (n = 3).

The effect of the extracts on keratinocyte migration was assessed using a scratch assay. Witches’ broom extract markedly reduced scratch closure in HaCaT cells, whereas the healthy birch extract showed a weaker effect under the same conditions (Figure 4). After 48 h of incubation, migration was inhibited by 99.09% ± 4.71% for witches’ broom extract compared to 84.33% ± 5.14% for healthy birch extract at the highest tested concentration compared with medium (control) (Table 2). Healthy birch extract showed a lower inhibitory effect, reaching approximately 80%–85% inhibition at the same concentration (Table 2).

**TABLE 2:** Effect of the tested extracts on cell migration assessed by scratch assay.

Concentration (µg/mL)	Witches’ broom extract (%)	Healthy birch extract (%)
Scratch closure after 4 h		
0 (Medium)	12.12 ± 4.82	
15.63	1.92 ± 0.45	3.86 ± 5.72
31.25	1.35 ± 2.45	1.20 ± 2.25
62.50	0.79 ± 1.71	0.35 ± 0.87
Scratch closure after 20 h		
0 (Medium)	29.72 ± 9.75	
15.63	18.63 ± 5.37	14.56 ± 8.93
31.25	9.15 ± 3.94	8.10 ± 4.41
62.50	0.24 ± 1.34	6.03 ± 5.85
Scratch closure after 28 h		
0 (Medium)	73.14 ± 30.22	
15.63	23.37 ± 7.45	24.31 ± 10.39
31.25	11.54 ± 4.66	13.34 ± 7.02
62.50	0.55 ± 2.50	11.33 ± 3.51
Scratch closure after 48 h		
0 (Medium)	95.36 ± 6.42	
15.63	28.43 ± 7.89	29.26 ± 9.02
31.25	12.12 ± 4.17	18.47 ± 8.03
62.50	0.87 ± 0.93	14.96 ± 4.90

The percentage of scratch area closure was measured after 4, 20, 28 and 48 h of incubation with the tested extracts at concentrations of 15.63, 31.25 and 62.5 µg/mL. Cells cultured in medium without the compound (0 µg/mL) served as the control. Results are presented as mean ± SD (n = 3).

### 3.4 IL-6 and IL-8 cytokine secretion

**FIGURE 5:**
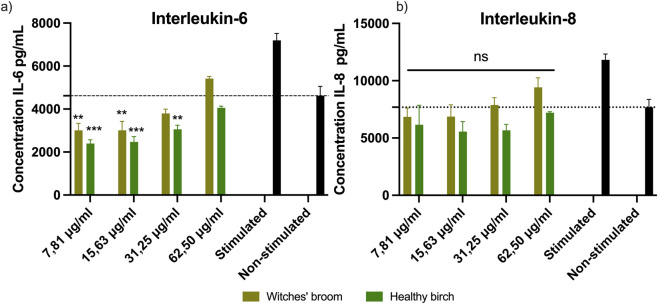
Dose-dependent effect of witches’ broom and healthy birch extracts on **(a)** interleukin-6, and **(b)** interleukin-8 secretion. Cells were treated with different concentrations of both extracts, the interleukin levels (pg/mL) were measured by ELISA. Data are presented as mean ± SEM from two independent experiments (n = 2). Statistical analysis was performed using one-way ANOVA. Significance vs. non-stimulated control: ns p > 0.05 – not significant; **p < 0.01 – significant; ***p < 0.001 – highly significant.

The effect of witches’ broom extract on IL-6 and IL-8 secretion was evaluated in HaCaT cells (Figure 5). Treatment with the extract resulted in a reduction of IL-6 level compared with stimulated control cells. The decrease in IL-6 concentration was significant at concentrations 7.81–31.25 µg/mL (Figure 5a). No statistically significant differences were observed for IL-8 at any concentration (Figure 5b).

### 3.5 Antimicrobial activity

Antimicrobial activity of the extracts was assessed using the disc diffusion method. Witches’ broom extract moderately inhibited the growth of *C*. *acnes*, producing an inhibition zone of 12 mm in diameter, whereas no measurable inhibition zones were observed for the remaining tested pathogens. The healthy birch extract did not produce detectable inhibition against any of the tested microorganisms. The positive controls (ciprofloxacin, amphotericin B) exhibited clear zones of growth inhibition, confirming that all strains were susceptible under the assay conditions (Table 3).

**TABLE 3:** Antimicrobial activity of witches’ broom and healthy birch extracts (1 mg/mL) determined by disc diffusion assay.

​	Inhibition zones			​
Strain	Witches’ broom extract	Healthy birch extract	Ciprofloxacin	Amphotericin B
*Candida albicans* MK25	6 mm	6 mm	—	23 mm
*Cutibacterium acnes* ATCC 11827	12 mm	6 mm	41 mm	—
*Escherichia coli* ATCC 25922	6 mm	6 mm	32 mm	—
*Micrococcus luteus* ATCC 10240	6 mm	6 mm	31 mm	—
*Staphylococcus aureus* ATCC 29231	6 mm	6 mm	26 mm	—
*Staphylococcus epidermidis* ATCC 12228	6 mm	6 mm	28 mm	—

Inhibition zones were measured against common skin pathogens. Results are presented as mean inhibition zone diameter (mm) from three independent experiments (n = 3). Ciprofloxacin (5 µg/disc) and amphotericin B (20 µg/disc) were used as a positive control. A value of 6 mm indicates no activity.

## 4 Discussion

The present study investigated the phytochemical composition and biological activity of aqueous extracts obtained from birch witches’ broom in comparison with extracts derived from healthy birch tissue. The results demonstrated qualitative differences in the phytochemical profiles of the two materials and revealed distinct effects on keratinocyte viability, migration, and cytokine secretion. In particular, witches’ broom extract showed a stronger effect on keratinocyte scratch closure and a more pronounced reduction of pro-inflammatory cytokine secretion than the extract prepared from healthy birch tissue. These findings support the hypothesis that infection-induced metabolic reprogramming of birch tissues results in the accumulation of biologically active compounds capable of modulating cellular processes relevant to skin physiology (Ullah et al., 2019; Smiljanic et al., 2022).

Phytochemical analysis revealed the presence of catechin-based compounds, hydroxybenzoic acids, arylbutanoids, diarylheptanoids, iridoids, hydroxycinnamic acids, and organic acids that did not fall into any of the previously mentioned categories. Previous phytochemical investigations of *Betula* species have reported similar classes of secondary metabolites, including quercetin derivatives, phenolic acids, and triterpenoids such as betulin and betulinic acid (not detected in the present chromatograms, likely due to their low water solubility), as well as diarylheptanoids (Smiljanic et al., 2022; Liimatainen, Karonen and Sinkkonen, 2012; Lee et al., 2012). These compounds are known to contribute to the antioxidant, anti-inflammatory, and cytotoxic properties of birch-derived materials (Ullah et al., 2019; Hayakawa et al., 2020; Chen et al., 2022).

The altered chromatographic profile observed for witches’ broom compared with healthy birch tissue suggests that fungal infection by *T. betulina* induces metabolic changes in host tissues (Ullah et al., 2019). Pathogen infection is known to affect plant hormone balance, carbon allocation, and defense-related pathways, often resulting in increased production of phenolic compounds and other secondary metabolites involved in stress responses. In the case of witches’ broom, the formation of abnormal, hypertrophic tissues is accompanied by profound physiological and biochemical alterations, which may explain the differences in metabolite composition detected in this study. Importantly, these infection-associated metabolites cannot be assigned unequivocally to either plant or fungal origin based on the present dataset. Most annotated compounds belong to metabolite classes previously reported in *Betula* species, supporting a substantial contribution of host-plant metabolism. However, because the extracts were prepared from whole infected tissue and no separate fungal mycelium extract was analyzed, some signals may also derive from fungal metabolites or host-pathogen co-metabolism. For this reason, the compounds discussed here are described as infection-associated metabolites rather than exclusively birch- or fungus-derived constituents.

Moreover, diarylheptanoids, which were detected in witches’ broom extracts, have been previously described as characteristic metabolites of birch species and are associated with diverse biological activities (Lee et al., 2012). Their presence in infected tissues may reflect either enhanced biosynthesis in response to fungal stress or altered degradation pathways. Taken together, these results indicate that witches’ broom represents a metabolically distinct plant material rather than a simple morphological anomaly, supporting its potential relevance as a source of bioactive compounds.

While the metabolomic profile supports witches’ broom as a chemically distinct birch-derived material with potential bioactivity, functional validation is essential to contextualize these differences in a biological setting. Therefore, we next evaluated the effects of witches’ broom and healthy birch extracts on the viability of skin-relevant cell lines and used non-cytotoxic concentrations for subsequent mechanistic assays. The cell viability assay (MTT) demonstrated concentration-dependent cytotoxic effects of both witches’ broom and healthy birch extracts on HaCaT and HTB-140 cell lines. The greater sensitivity of HaCaT keratinocytes compared with HTB-140 melanoma cells indicates that the extracts exert stronger effects on non-malignant epidermal cells than on the tested melanoma line under the present experimental conditions. This finding should be interpreted cautiously and primarily as a safety-related observation rather than as evidence of therapeutically favorable selectivity. In particular, the lack of preferential toxicity toward melanoma cells argues against an antineoplastic interpretation, while the higher keratinocyte sensitivity underscores the need for careful dose selection in future dermatological applications to avoid compromising epidermal barrier integrity. For this reason, subsequent functional assays were restricted to concentrations that did not significantly reduce HaCaT viability relative to untreated controls. To our knowledge, no MTT-based cytotoxicity data have previously been reported for *Taphrina*-induced galls; thus, this study provides the first such evidence for *T. betulina* witches’ broom. However, our results are consistent with previously reported cytotoxic activity of birch-derived extracts towards skin cells, often linked to polyphenolic disruption of cell membranes or pro-apoptotic signaling (Smiljanic et al., 2022; Adepoju et al., 2023). Comparable MTT-based cytotoxic effects have also been described for other infection-induced plant pathologies, such as tree galls, which are known to accumulate phenolic compounds (Yosefsani et al., 2023; Wan Nur Suzilla Wan and Hasmah, 2020; Shankara et al., 2016).

Next, we assessed functional impacts on skin physiology, focusing on keratinocyte functional responses (viability and scratch closure) - a central determinant of epidermal homeostasis and dysregulated in inflammatory skin conditions such as psoriasis and atopic dermatitis. By defining non-cytotoxic threshold, we were able to evaluate the extracts’ functional impact on skin physiology at concentrations that do not compromise cellular integrity. In the scratch assays, witches’ broom extract reduced the rate of scratch closure compared with the control and healthy-tissue extract, indicating altered keratinocyte behavior under these conditions. The reduced scratch closure should be interpreted as altered keratinocyte motility and/or proliferative capacity as this assay integrates both migration and proliferation of the cells. Specifically, at the highest non-toxic concentration tested (62.5 µg/mL), the witches’ broom extract maintained nearly total inhibition of scratch closure by approximately 99%, from the 20^th^ to the 48^th^ hour of observation. In contrast, the healthy birch extract at the same concentration exhibited a lower inhibition rate of 80%–85%. This effect suggests that fungal infection does not merely preserve baseline birch bioactivity but can shift the functional phenotype of the extract - consistent with the metabolomic evidence that witches’ broom represents a chemically distinct plant material. This shift could arise from increased accumulation of specific phenolics (including flavonoid derivatives and diarylheptanoids) as part of a stress-associated defense response, or from infection-driven changes in metabolite turnover that favor compounds with greater bioactivity in skin models. Because these experiments were performed within the non-cytotoxic range defined by MTT, the reduced gap closure is unlikely to reflect nonspecific loss of viability and instead suggests a targeted modulation of motility-related pathways. However, strong inhibition of scratch closure should not be interpreted as universally beneficial in dermatology: while it could be disadvantageous in the context of acute wound repair by delaying re-epithelialization, it may be relevant in hyperproliferative skin disorders such as psoriasis, where excessive keratinocyte turnover and inflammatory remodeling contribute to disease pathogenesis.

Building on the observed effects on scratch closure, we examined witches’ broom extract’s impact on inflammatory signaling, as dysregulated cytokine production contributes to skin disorders such as atopic dermatitis and psoriasis. Witches’ broom extract significantly reduced IL-6 secretion in TNF-α/IFN-γ-stimulated HaCaT cells, while IL-8 showed no significant change under tested conditions. Similar anti-inflammatory effects are reported for birch phenolics and related phytochemicals that modulate cutaneous inflammatory responses (Guo and Dipietro, 2010; Liimatainen, Karonen and Sinkkonen, 2012; Xiang et al., 2019). Similarly, Popowski et al. demonstrated anti-inflammatory activity of *Betula pendula* leaf infusion, including modulation of IL-6 and IL-8 secretion, and provided evidence of bioavailability and biotransformation of phenolics in humans (Popowski et al., 2024). The enriched procyanidins and diarylheptanoids in witches’ broom may contribute to the observed IL-6-lowering effect, as these classes suppress Th17-related cytokines in skin inflammation models. However, reactive oxygen species generation, apoptosis markers, and signaling pathways (e.g., NF-κB, MAPK) were not assessed. Therefore, the mechanism underlying the cytokine response cannot be defined based on the current data and should be regarded as hypothesis-generating rather than mechanistically resolved. Importantly, the non-cytotoxic concentration range used in downstream assays (≤62.5 µg/mL, >80% viability in MTT) likely reflects a biologically relevant, non-cytotoxic exposure window, at least in terms of cellular tolerance and functional responsiveness. However, the translational relevance of these findings requires confirmation in more complex models, including 3D skin equivalents and dermal penetration studies.

Disc diffusion revealed moderate *C. acnes* inhibition by witches’ broom (12 mm zone), absent for other pathogens and healthy birch. Although the antimicrobial assessment was limited to an initial disc diffusion screen, this selective activity against acne-associated anaerobes–rather than broad-spectrum effects–may be particularly advantageous for dermatological applications by preserving skin microbiota balance. This specificity likely links to phenolic enrichment, distinguishing witches’ broom from triterpene-dependent birch extracts (Blondeau et al., 2020). The use of aqueous extraction likely limited broader antimicrobial activity of aqueous birch extracts as previously reported (Cushnie and Lamb, 2011). Overall, witches’ broom exhibits chemically and functionally distinct skin-modulating properties, including immunomodulatory effects, strong inhibition of scratch closure, and limited but potentially selective anti-*C. acnes* activity. While these findings are consistent with the Sámi traditional use of witches’ broom, the biological significance of the anti-migratory effect is likely context-dependent rather than universally beneficial.

## 5 Conclusion

The present study provided a phytochemical and biological characterization of witches’ broom extract obtained from birch trees in comparison with an extract derived from healthy birch material. Distinct differences in phytochemical profiles were observed between the two extracts, which may reflect metabolic alterations associated with fungal infection by *T. betulina* Rostr (1883) (Christita, Auzane and Overmyer, 2023; Christita and Overmyer, 2021).

Both extracts exhibited concentration-dependent effects on human keratinocyte (HaCaT) and human melanoma (HTB-140) cell viability (van Meerloo, Kaspers and Cloos, 2011; Mazloum-Ardakani et al., 2019), as well as an inhibitory influence on keratinocyte scratch closure (Tampa et al., 2022; Smiljanic et al., 2022). In addition, witches’ broom extract reduced IL-6 secretion in stimulated keratinocytes, while showing no significant effect on IL-8 under the tested conditions. Finally, witches’ broom extract exhibited moderate activity against *C. acnes* – a key pathogen in acne vulgaris – while healthy birch extract showed no activity against any tested strains. Taken together, these findings indicate that witches’ broom extract contains bioactive compounds capable of modulating cellular processes relevant to skin physiology, particularly cell viability, scratch closure dynamics, and inflammatory responses. While strong inhibition of keratinocyte migration may be unfavorable in the context of acute wound healing, it may be relevant in hyperproliferative disorders such as psoriasis, where excessive keratinocyte turnover contributes to disease pathogenesis. Although these effects were demonstrated only *in vitro* and require further validation, the results position witches’ broom as a promising source for future compound isolation and targeted dermatological research.

## Data Availability

The original contributions presented in the study are included in the article/supplementary material, further inquiries can be directed to the corresponding author.
